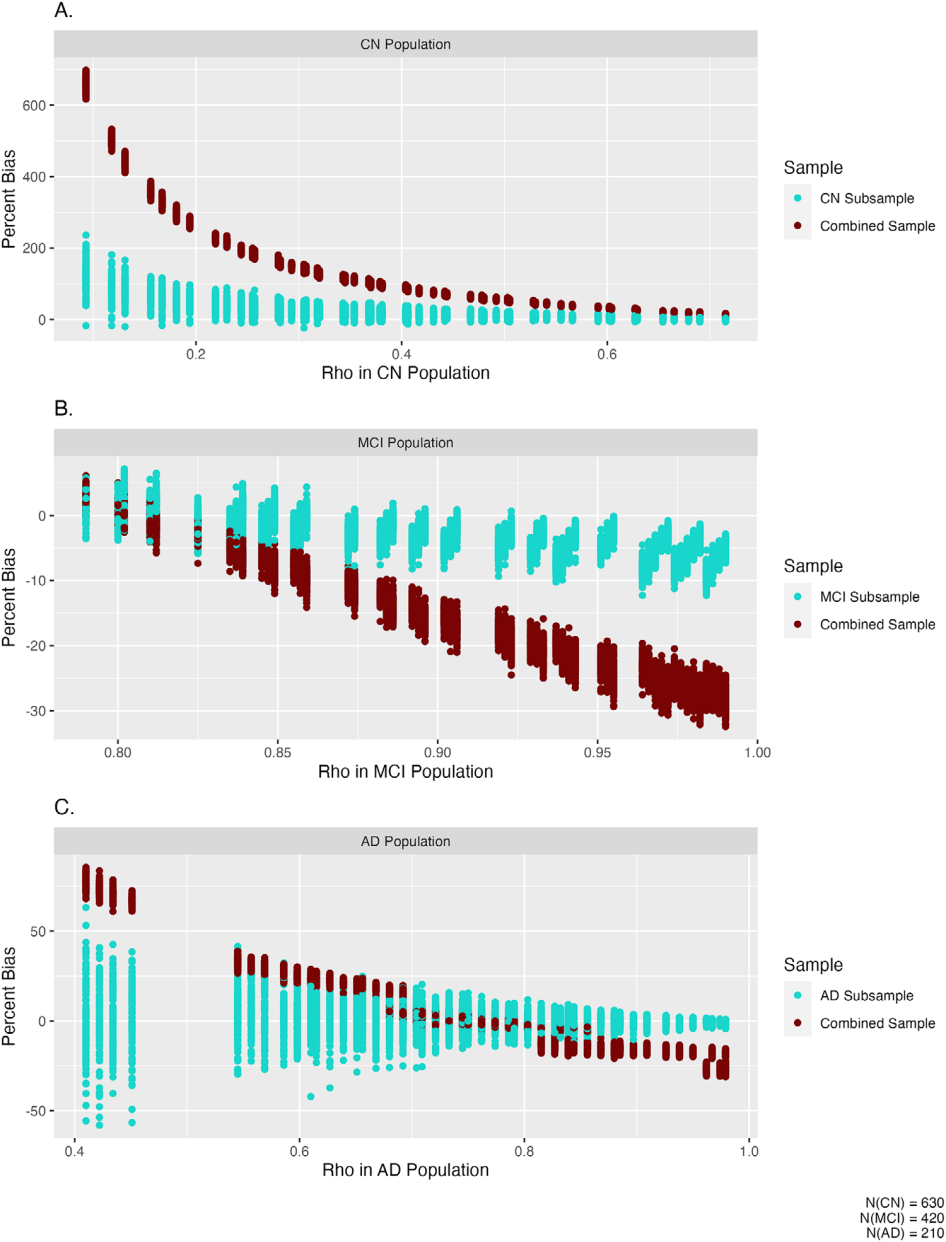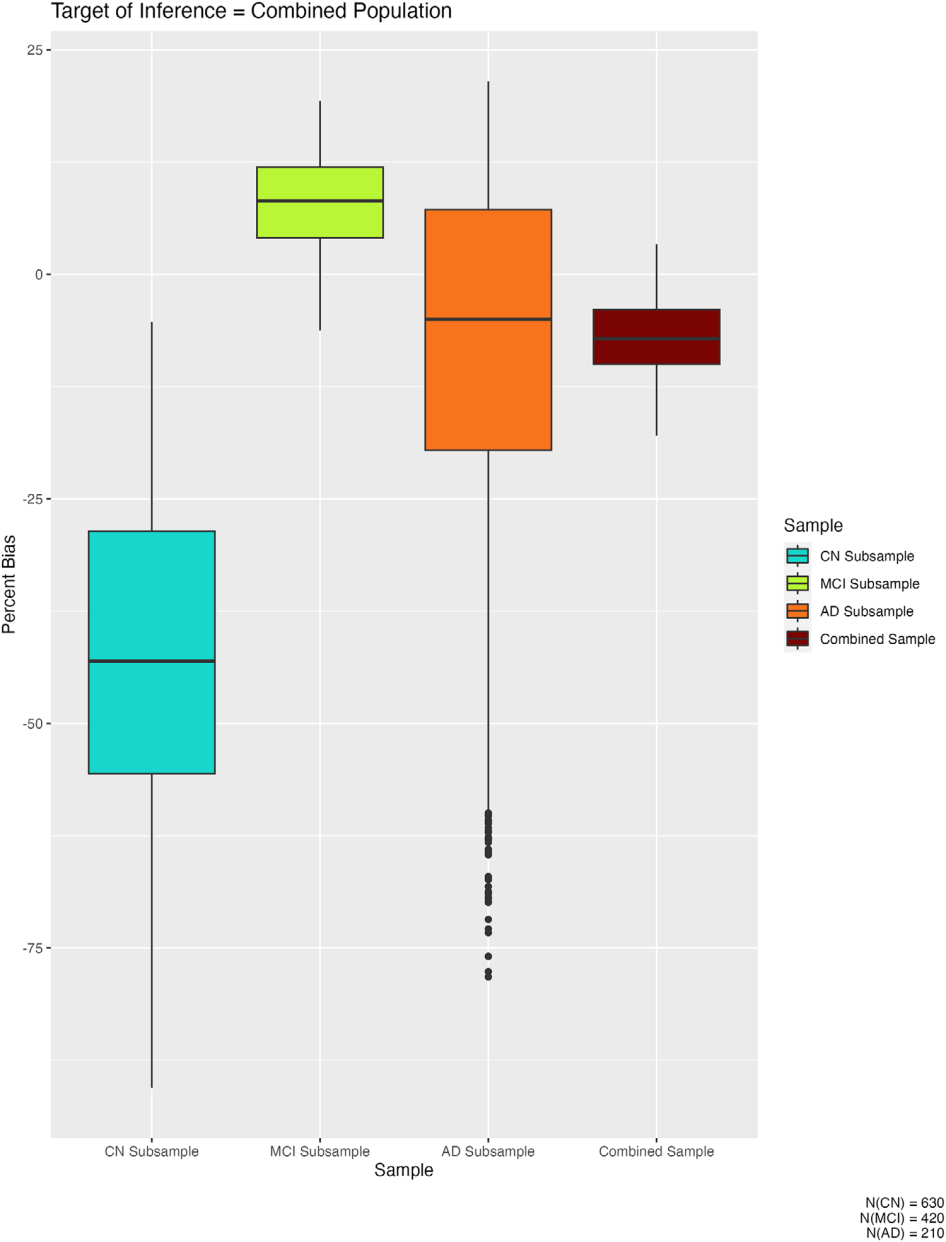# Bias in estimating brain‐cognition associations by clinical diagnosis: A simulation study

**DOI:** 10.1002/alz.087815

**Published:** 2025-01-03

**Authors:** Brandon E Gavett, Evan Fletcher, Keith F. Widaman, Sarah Tomaszewski Farias, Dan M. Mungas

**Affiliations:** ^1^ University of California Davis, Sacramento, CA USA; ^2^ UC Davis, Davis, CA USA; ^3^ University of California at Riverside, Riverside, CA USA; ^4^ University of California, Davis, Sacramento, CA USA

## Abstract

**Background:**

Researchers often divide heterogeneous samples into homogeneous subgroups for analysis. For example, a sample of participants with normal cognition, mild cognitive impairment (MCI), and dementia might be divided into subgroups to answer questions about whether some exposure variable (e.g., brain atrophy) predicts some outcome variable (e.g., cognitive decline) differently across groups. It is unclear, however, whether subgroup analysis provides greater insight into the associations between neurodegeneration and cognitive decline, or whether it is counterproductive. The current study employed statistical simulation to address this issue.

**Methods:**

Using data from the Alzheimer’s Disease Neuroimaging Initiative, we estimated the correlation between gray matter volume slopes and episodic memory slopes. This correlation (r = .813) and subgroup descriptives formed the population parameters upon which simulations were based. We generated plausible scenarios where the correlations in the three subgroups could differ while maintaining the same overall correlation in the combined sample. Diagnostic subgroups were derived by simulating a global CDR score (0 cognitively normal; 0.5 MCI; 1+ dementia) for each simulated case. We also manipulated the relative sample size differences between the three groups. The primary outcome variable was the amount of bias (difference between simulated sample correlation and known population correlation) in estimating the strength of association between gray matter change and cognitive change. Samples were (1) combined and (2) analyzed separately by diagnostic group.

**Results:**

Estimates specific to a diagnostic subgroup were least biased when the analyses were limited to samples drawn from that population (e.g., an MCI sample to make inferences about the MCI population). In contrast, when making inferences about the entire spectrum of cognitive aging, more nuanced findings appeared. In general, the least bias was observed in the combined sample, but this was only true when cognitively impaired cases were adequately represented.

**Conclusion:**

Decisions about whether to stratify analyses by diagnostic subgroup require consideration of the target of inference. If the goal of cognitive aging research is to understand the cognitive manifestations of neurodegeneration across the full spectrum of disease, then subgroup analysis is likely to be more biased than the combined sample. With representative samples, both approaches may provide complementary information.